# Introduction

**DOI:** 10.3402/jmahp.v3.29204

**Published:** 2015-08-12

**Authors:** Mondher Toumi, Walter Ricciardi

**Affiliations:** 1 Faculty of Medicine, Public Health Department, Research Unit EA 3279, University Aix-Marseille, Marseille, France; 2 European Public Health Association, Department of Public Health, Catholic University of the Sacred Heart, Rome, Italy

It is hard to estimate the value of vaccines. They have contributed substantially to the reduction of burden from communicable diseases and associated mortality. Each year, three million lives are saved, thanks to vaccination (1, 2). In developed countries, routine vaccination has led to complete eradication or control of several infectious diseases (3).

This special issue of the *Journal of Market Access and Health Policy* is dedicated to vaccines and consists of a series of seven articles that present undeniable and robust evidence on benefits of vaccination from an economic perspective. We consider it extremely important to highlight these benefits in order to retain public trust in vaccination and to keep and strengthen the reliability of immunisation policies and programmes. All the seven articles give a broad overview of several aspects related to immunisation:

- They depict the impact of vaccination on economic growth, sustainability, and efficiency of healthcare systems. It has been shown that economic growth is driven by improved health (4–6). Vaccination is recognised as a substantial preventive measure that improves health and allows individuals to contribute to economic growth by better physical, cognitive, and educational performance (7). Immunisation keeps people healthy and ensures retention of healthcare resources (8). Vaccination is one of the most cost-effective interventions that contribute to healthcare system efficiency (9, 10).
- They show the true economic and societal value of vaccination. There are several intangible gains provided by vaccination that are ignored by traditional economic analyses. These include outcome-related productivity gains (improved cognition and physical strength, as well as school enrolment, attendance, and attainment), behaviour-related productivity gains (influence on fertility and consumption choices), and community externalities (herd effect, indirect protection, prevention of antibiotic resistance) among others (11, 12).
- They also discuss short- and long-term benefits that can be obtained with vaccination in the context of relatively low levels of investments. Several examples demonstrate that, apart from commonly recognisable long-term gains, vaccines are also able to provide short-term benefits, which result in rapid returns on investments (13–18).
- Finally, they give ideas on actions that need to be taken by governments, international agencies, and other stakeholders, such as the medical community, in order to make benefits of vaccination programmes fully recognised and appreciated.

The present series constitutes a comprehensive source of information on the benefits of vaccines and depicts the usefulness of vaccines from different angles and perspectives. They help realise the broad spectrum of benefits that add to health, and of the economic and societal gains of immunisation. Conveying this message to the patients and authorities, thereby ensuring increased awareness of the true value of vaccination, is of great importance in today's world in Europe where vaccine's hesitancy and underuse may lead to severe outbreaks, as recently observed in Germany, with measles outbreaks, and in Spain, where one case of diphtheria was registered for the first time since 1987. Promoting and strengthening the role of vaccines is in line with a document recently published by the Council of the European Union (19). The council calls member states to continue to develop comprehensive and coordinated approaches to vaccination programmes and to advocate and encourage the use of vaccines.

*Mondher Toumi*, Editor-in-Chief Faculty of Medicine, Public Health Department
Research Unit EA 3279, University Aix-Marseille
Marseille, France *Walter Ricciardi*
European Public Health Association
Department of Public Health
Catholic University of the Sacred Heart
Rome, Italy
